# New Anticancer Agents: Design, Synthesis and Evaluation

**DOI:** 10.3390/ijms26136090

**Published:** 2025-06-25

**Authors:** Vadim Pokrovsky, Raffaele Addeo, Mauro Coluccia

**Affiliations:** 1 Laboratory of Combined Treatment, N.N. Blokhin National Medical Research Center of Oncology of Ministry of Health of Russian Federation, 115478 Moscow, Russia; v.pokrovsky@ronc.ru; 2 Medical Oncology Unit, San Giovanni di Dio Hospital, 80027 Naples, Italy; raffaeleaddeo19@gmail.com; 3 Department of Pharmacy-Drug Sciences, University of Bari “Aldo Moro”, Via E. Orabona 4, 70125 Bari, Italy

Among the fundamental pathological processes, tumorigenesis is arguably the most complex. It results from the accumulation of genetic alterations that typically unfold over many years, leading to the gradual breakdown of homeostatic barriers at the cellular, tissue, and ultimately organismal levels. Over the past few decades, discoveries on the molecular and cellular mechanisms of tumorigenesis have had, and continue to have, a profound impact on anticancer pharmacotherapy. It is noteworthy that since the early 2000s, the number of anticancer drugs approved for clinical use has increased from a few dozen [1] to several hundred distinct agents [2]. With the aim of targeting the hallmarks of tumorigenesis [3]—the functional traits that enable cancer cells to survive, proliferate, and disseminate—numerous classes of anticancer drugs are currently under active investigation and development. This Special Issue on “New Anticancer Agents: Design, Synthesis and Evaluation” brings together experimental studies and review articles that, at least in part, reflect the complexity of this disease and highlight several aspects of basic research aimed at therapeutic innovation.

The immune system serves as a protective barrier that can restrain tumorigenesis until conditions within the tumor tissue enable tumor cells to acquire an immune-evasive phenotype. Avoiding immune destruction is therefore considered a core hallmark of cancer [3], and the upregulation of immune checkpoint molecules (PD-1, CTLA-4, LAG-3) is a well-characterized mechanism of immunoevasion [4]. While monoclonal antibodies (mAbs) currently remain the primary form of immune checkpoint inhibitors (ICIs) in clinical practice, non-mAb ICIs are an exciting and growing area of research. In the paper by Gil-Edo et al. [5], the authors report the synthesis of a series of triazole derivatives bearing electron-withdrawing or electron-donating groups at the R position on a phenyl ring, combined with either small polar groups (OH, P=H) or a bulky lipophilic group (4,4′-dimethoxytrityl). The pharmacological activity of these derivatives, which are inspired by Holak’s pharmacophore model of PD-1/PD-L1 inhibitors [6], was evaluated on HT-29, MCF-7, and A-549 cancer cell lines. The compounds under investigation did not inhibit the growth/proliferation of tumor cells in (mono)culture; however, depending on their structural characteristics and the tumor cell type, they inhibited the expression of PD-L1 and c-MYC, particularly in MCF-7 and HT-29 cells. This effect was related to the ability, more pronounced in some derivatives, to sensitize tumor cells to the suppressive action of Jurkat T or THP-1 immune cells in co-culture systems, thus suggesting a potential immunomodulatory mechanism of selected triazole derivatives.

Nitrogen-containing heterocycles, including imidazoles, fused imidazole systems, and triazole derivatives, constitute a pivotal scaffold class in medicinal chemistry, being extensively employed in the design of enzyme inhibitors, receptor ligands, and antimicrobial agents. According to Vitaku E. et al. [7], nearly 60% of the small molecule drugs approved by the U.S. FDA consist of nitrogen-containing heterocycles. Within this class, the most frequently utilized molecules, imidazole and fused imidazoles, have been shown to interfere with various tumor targets, and their key features of structure–activity relationships have been described [8]. In the study by Lee et al. [9], the design and synthesis of novel 1,4-dialkoxynaphthalene-based imidazolium salts are presented, along with the evaluation of their in vitro growth inhibition activity against human cancer cell lines. The results allowed for the establishment of an initial, qualitative structure–activity relationship based on observations of activity changes corresponding to structural modifications. Interestingly, the 1,4-dialkoxynaphthalene-based imidazolium salts were synthesized using a novel method, which was subsequently employed by the same group to produce new derivatives capable of inhibiting the ERK5 kinase [10].

The 4-thiazolidinone scaffold is an attractive structural framework in anticancer drug research, both for its structural flexibility and for the extensive experimental evidence demonstrating its ability to simultaneously inhibit multiple cellular pathways involved in tumorigenesis [11]. The review by Roszczenko et al. [12] integrates recent studies on 2-thioxothiazolidin-4-one (rhodanine) [13] and thiazolidine-2,4-dione [14], and summarizes data on new 4-thiazolidinone-based hybrid molecules with potential anticancer activity reported between 2017 and 2022. It highlights key trends in molecular hybridization techniques, including scaffold hybridization, the hybrid pharmacophore method, and analog-based drug design of 4-thiazolidinone cores, incorporating early-approved drugs, natural compounds, and privileged heterocyclic scaffolds. The primary focus is on utilizing these strategies to design small molecules with anticancer potential. Overall, the review enriches the structure–activity relationship (SAR) profile of 4-thiazolidinone hybrids, providing valuable insights for the design, optimization, and development of new antitumor compounds.

Selectively targeting tumor tissue and cells is a pivotal concept in anticancer pharmacotherapy, especially in the development of targeted drugs, nanomedicines, and imaging agents. Aimed at selectively targeting tumor cells while minimizing collateral damage, Directed Enzyme Prodrug Therapy (DEPT) is based on the administration of a prodrug that is locally activated by a specific enzyme [15]. Choosing the suitable enzyme [16,17] and achieving an adequate enzyme concentration in the tumor tissue is one of the critical aspects of DEPT [18]. The enzyme can be directed to the tumor site through various strategies, such as conjugation with tumor-specific carriers, as shown in the work of Pokrovsky’s group [19]. This research team had previously shown that the enzyme methionine γ-lyase (MGL) was capable of catalyzing the breakdown of S-substituted L-cysteine sulfoxides (propiin), leading to the formation of thiosulfinates with cytotoxic activity [20]. Thiosulfinates, particularly the more stable derivatives of the parent compound allicin, are currently considered promising anticancer agents [21]. By utilizing a mutant form of the enzyme with enhanced efficiency [22], conjugated with the phytoestrogen daidzein [23] to promote tumor targeting, the team also demonstrated promising in vitro and in vivo activity against breast cancer cells [24]. In this study, the antitumor activity of the pharmacological pair MGL–Daidzein/S-propyl–L-cysteine sulfoxide was evaluated on human tumor cells representative of colon, pancreas, and prostate cancers, showing variable cytotoxic potency in vitro, and the effective inhibition of tumor growth in vivo, presumably associated with the expression of daidzein receptors on the tumor cells.

The vaterite polymorph of calcium carbonate is being increasingly used as a biocompatible carrier in a range of biomedical applications, particularly in drug delivery and controlled release systems [25]. Examples of anticancer applications include vaterite-CaCO3 nanoparticles loaded with doxorubicin [26], 5-fluorouracil [27], and cisplatin [28]. In the study by Niza-Perez et al. [29], vaterite-phase CaCO_3_ nanoparticles were functionalized with L-cysteine and manganese with the aim of linking the cysteine dependence of certain tumor subtypes, particularly glioblastoma, to the cytotoxic activity of manganese. A comprehensive set of characterization techniques—including infrared and UV-visible spectroscopy, X-ray diffraction, X-ray fluorescence, and transmission electron microscopy—confirmed the successful functionalization of the vaterite nanoparticles. Preliminary in vitro investigations of the cytotoxic effects of the vaterite-based materials were performed on murine glioma and human breast cancer cell lines, with the results encouraging further evaluation in in vivo models.

Targeting cancer metabolism is a promising and increasingly sophisticated strategy that leverages the distinct energy and biosynthetic demands of cancer cells [30]. Nicotinamide adenine dinucleotide (NAD^+^) is a pivotal molecule in cellular metabolism, influencing various aspects of cancer biology, e.g., energy production, DNA repair, and cell survival, making its metabolism a critical factor in tumor progression and therapy responses [31]. Accordingly, NAD^+^-producing enzymes have been widely investigated as potential targets. In their review [32], Ghanem et al. summarize the role of NAD^+^ in cancer metabolism and NAD^+^ biosynthetic pathways in mammalian cells. The relevance of NAD^+^-producing enzymes as potential targets in cancer is widely illustrated, with particular regard for the reported molecules apart from nicotinamide phosphoribosyltransferase (NAMPT) that inhibit the activity of NAD^+^-producing enzymes [33]. In addition, the potential challenges that might be encountered in the employment of these inhibitors as drug candidates in oncology are also highlighted.
